# Indigenous Knowledge about Consumption of Edible Insects in South Africa

**DOI:** 10.3390/insects12010022

**Published:** 2020-12-31

**Authors:** Zabentungwa T. Hlongwane, Rob Slotow, Thinandavha C. Munyai

**Affiliations:** School of Life Sciences, University of KwaZulu-Natal, Private Bag x 01, Scottsville 3209, South Africa; Slotow@ukzn.ac.za (R.S.); munyaic2@ukzn.ac.za (T.C.M.)

**Keywords:** edible insects, entomophagy, nutritional benefits, mopane worm, termites

## Abstract

**Simple Summary:**

Edible insects are a natural resource rich in protein, fats, vitamins, amino acids, and minerals such as zinc and iron. Consumption of insects is a traditional practice in many African countries. Edible insects are consumed for their nutritional value and play an essential role in human nutrition across Africa. We conducted questionnaires intending to document indigenous knowledge regarding the consumption of insects, and collection and preparation methods used by rural people in Limpopo and KwaZulu-Natal (KZN), provinces of South Africa. We found that most people still consume insects in Limpopo while fewer people do so in KZN. In KZN, respondents cited that the decline in entomophagy might be caused by the adoption of western diets, discomfort associated with eating insects, and the decline in the availability of insects from the wild. Mopane worms and termites were the most preferred insects in Limpopo and KwaZulu-Natal, respectively. Edible insects contribute to human nutrition and play an important role in improving the livelihoods of people in rural areas of South Africa. As a result, people should be encouraged to include edible insects in their daily diets.

**Abstract:**

Consumption of edible insects is an indigenous practice that has played an essential role in human nutrition across Africa. The traditional use of insects forms an important part of food culture in Africa, and insects are consumed either as a delicacy, emergency, or staple source of food. However, indigenous knowledge about insect consumption is being lost because recent generations have adopted western methods and paid less attention to traditional practices. We conducted 500 questionnaires in five local municipalities in Kwazulu-Natal (KZN), and 122 questionnaires in four local municipalities in Vhembe district in Limpopo, South Africa, to document indigenous knowledge about edible insects’ consumption, collection, and preparation methods used in Limpopo and KZN. Eight insect species belonging to five insect orders were used as food in Limpopo and KZN, with mopane worms (94%) and termites (70%) being the most preferred species by respondents in Limpopo and KZN, respectively. Ninety-five percent of the respondents occasionally consumed insects in Limpopo, while only 28% did so in KZN. Nutritional benefits and tradition were the main reasons for consuming insects. Edible insects are a nutritious diet and play an important role in people’s livelihoods in rural areas. However, there was a notable decline in entomophagy, particularly in KZN. The decline may be related to occidental acculturation, discomfort associated with eating insects, and declining insect availability. To promote entomophagy, the authorities should encourage people to include edible insects in their diets because of their nutritional value. In addition, edible insect flour should be incorporated in food products such as biscuits, bread, energy bars, cereal, and cookies to promote acceptability.

## 1. Introduction

Entomophagy is an ancient indigenous practice that has played a significant role in human nutrition around the world [1,2]. Edible insects are an important protein source, and their consumption plays an important role in food security and improving rural livelihoods [2,3,4,5]. They are consumed as a traditional food in different regions, including Latin America [6], China [7,8], Thailand [1,9], Mexico [10,11], Japan [12,13], and Africa [14,15,16,17,18,19,20,21,22]. The traditional use of insects as food in these countries is not a new practice, as it dates back as far as the history of mankind [10,11,12]. However, consumption of insects is not well accepted in western countries and still remains unexplored, because of barriers such as fear and disgust associated with eating insects [23,24,25,26]. In addition, in developed countries, eating insects is considered primitive, unclean, or food of the poor [24]. In Africa, approximately 500 species of insects are used as food in different countries [5,27]. On this continent, edible insects are consumed either as a staple, an emergency food source during times of food shortage, or an important delicacy [22,28]. Consumption and preference of edible insects differ geographically [22]. For example, some people prefer consuming insects, either fried, roasted, or raw, and others may find eating insects disgusting [5,14]. In some African countries, certain species are only consumed in one region and are of traditional importance; for example, stink bugs (Hemiptera: Tessaratomidae) are only consumed and are a delicacy for the vhaVenda people in the Limpopo province of South Africa [15].

In South Africa, edible insects have formed part of the human diet since prehistoric times; for example, Ledger [29] reported that South Africans consumed *Trinervitermes trinervoides* (a termite) and *Apis mellifera* unicolor (a honeybee) in early 100,000 BCE. In addition, Quin [20] and Bodenheimer [30] reported an ongoing consumption of edible insects for nutritional benefits by the baPedi people in South Africa in the early 1950s. Nowadays, in South Africa, edible insects are mostly consumed in Limpopo province by vhaVenda, baPedi, and Vatsonga people [13,15,31]. In addition, they are also consumed in Mpumalanga, KwaZulu-Natal [32], North West, and Gauteng [15]. The groups of edible insects consumed in South Africa are various Lepidopteran caterpillars, termites, grasshoppers, jewel beetles, ants, and stink bugs [13,15,29,33]. Edible insects are an important natural resource available to vulnerable people and provide earning opportunities to traders and harvesters [31,34,35,36,37]. In addition, edible insects play an important role in food security, rural livelihoods, and poverty eradication [34]. For example, Makhado et al. [33] reported that trading edible insects results in an income of approximately US$202,915 per trader during one season. Edible insects create seasonal employment opportunities for unemployed people across southern Africa, reducing poverty and improving human wellbeing [31,34].

Malnutrition and food shortage are the major challenges experienced in developing countries [38]. According to Voster [39], most people in South Africa are food-insecure and do not have access to nutritious food to meet their daily nutrient requirements. Edible insects are consumed because they are a nutritious traditional food source that has been used to supplement diets across Africa over many years [22]. In addition, consumption of insects is good for human wellbeing, as the nutritional value of edible insects can help promote human health, and reduce the vulnerability to malnutrition of children, pregnant women, and older people [40]. Edible insects can also improve the well-being of vulnerable people living with malnutrition, child stunting, and macronutrient deficiencies [41,42,43,44].

Over the years, there has been a notable decrease in entomophagy in developing countries, particularly in urban areas [4,21]. The younger generation, especially in urban areas, has little or no knowledge about insects’ consumption [4]. This is thought to result from people adopting western/modern food culture and, therefore, abandoning traditional practices such as entomophagy [4]. Globalization and westernization have influenced what people eat [23]. As a result, people are more reliant on western food, and they are more reluctant to try traditional food such as edible insects, as they are now perceived as dirt or a taboo [4,23,44]. This has led to several people being wary of accepting insects as food or even distancing themselves from consuming insects [4,23,45]. Yet, edible insects are rich in protein, carbohydrates, amino acids, and micronutrients, such as zinc and iron [5].

Little attention has been paid to documenting indigenous knowledge on insects’ consumption as an important traditional practice in South Africa [46,47,48]. The traditional understanding of entomophagy among indigenous people is rich but restricted because it is orally passed through generations, and some has been lost in translation over the years [1,49]. Mostly, rural communities have no tools or resources to document their indigenous knowledge about practices that play an important role in their communities. As a result, indigenous knowledge is lost because recent generations have adopted western methods and paid less attention to traditional practices [4,50]. Combining indigenous knowledge about entomophagy with scientific research will further improve the understanding of the role of edible insects as a food resource for people [49]. In addition, documenting indigenous knowledge about edible insects will help promote and preserve entomophagy [4]. The current study, therefore, aims to (1) document indigenous knowledge relating to the consumption patterns, methods, or techniques used in the collection, and preparation of insects in South Africa; (2) determine the most consumed or preferred insect species; (3) access the perception of edible insects; and (3) determine the factors influencing the consumption of edible insects in South Africa.

## 2. Materials and Methods

### 2.1. Study Site

The study was conducted in villages at KwaZulu-Natal and Limpopo provinces, South Africa. In KwaZulu-Natal, sampling was conducted in five villages (Swayimane, Umbumbulu, Nhlazuka, Tugela Ferry, and Kokstad) of the uMshwathi, eThekwini, Richmond, uMsinga and Greater Kokstad local Municipalities, while in Limpopo, sampling was conducted in several villages of the Mutale, Makhado, Thulamela, and Musina local Municipalities of Vhembe District Municipality (see Table A1). In the villages surveyed, the population size ranged from 86 to 4099 in Limpopo and 2000 to 7903 in KwaZulu-Natal (KZN) [51,52] (Table 1). These villages were selected because consumption of insects mainly forms part of their tradition. However, we are not undermining the other nearby or known villages in South Africa where edible insects’ consumption is also practiced.

### 2.2. Questionnaires

Questionnaires made up of closed and open-ended questions were conducted in KZN (*n* = 500) in August 2019 and Vhembe district municipality (*n* = 122) in December 2019, by visiting local peoples’ households to obtain their perspective on the subject of eating insects. The respondents were chosen randomly by approaching people door to door in their households (see Appendix B). The KZN sample was larger as there was more variability in answers, and the villages were more extensive and further apart. Questionnaires were conducted through face-to-face interviews. Questions assessed respondents’ knowledge about edible insects, ways of assessing insects, processing and preparing, reasons for eating insects, how often they consumed insects, the benefits of eating insects, and their attitude towards eating insects. In addition, respondents were asked to list the names of the insects consumed or used as food in their villages. Respondents were provided with pictures of different edible insects taken from the guide of insects of Southern Africa [53] and from the internet, to select the species consumed, or previously consumed, in their villages. In addition, as part of a separate study, we purchased edible insects from all the traders we interviewed in markets in Vhembe district, and these were used to identify the insects consumed. Questionnaires were written and presented by a first-language-speaking author using local languages isiZulu (KZN, ZTH) and Tshivenda (TCM) in Vhembe district of Limpopo. Demographic information of the respondents who participated in the study in both provinces are presented in (Table 2 and Table 3)

This study has been ethically reviewed and approved by the University of KwaZulu-Natal Human and Social Sciences Research Ethics Committee (approval number HSS/0125/019D). Permission to conduct research in various villages in all the local municipalities was obtained from community leaders. All participants provided informed consent to participate in the study, and data were anonymised, treated confidentially, and stored securely.

### 2.3. Data Analysis

Data from the questionnaires were coded and entered into an excel spreadsheet. A chi-square test of independence was used to determine if there were any significant differences in consumption pattern of edible insects across local municipalities. To determine if there were significant differences in factors influencing the choice to consume insects, a Generalized Linear Model (GLM) was used. Count data of respondents are presented in percentages. The GLM and chi-square test analysis were performed using IBM SPSS Statistics version 26 (SPSS Inc., Chicago, IL, USA).

## 3. Results

### 3.1. Insect Consumption Pattern

Eight species belonging to five insect orders were consumed as food in the Vhembe district and KZN (Table 4). In KZN, termites were consumed consistently (*X*^2^_4_ = 2.243, *p* = 0.619) at about 70% of respondents across the five villages, while edible locust (6–40%) and *Cirina forda* (0–19%) were consumed less, with significant differences across the villages (locust: *X*^2^_4_ = 59.313, *p* < 0.001; *Cirina forda*: *X*^2^_4_ = 44.457, *p* < 0.001) (Table 5).

Mopane worms were consumed consistently (*X*^2^_3_ = 1.664, *p* > 0.05) at about 94% of the respondents across villages in Limpopo, followed by termites (85%) and edible grasshoppers (84%) with no significant differences across villages (termites *X*^2^_3_ = 12.475, df = 3, *p* < 0.05; 11.407, df = 3, *p* < 0.05; edible grasshopper *X*^2^_3_ = 11.990, df = 3, *p* < 0.05), while *Encosternum delegorguei* (stink bug) (19%), *Gynanisa* caterpillar (18%), *Carebara vidua*. (15%), and *Cicadoidea* (1%) were consumed less, with no significant differences across villages (stink bug: *X*^2^_3_ = 3.398, *p* > 0.05); lepidopteran caterpillar: (*X*^2^_3_ = 1.406, 3*p* > 0.05), *Carebara vidua* (*X*^2^_3_ = 0.72, *p* > 0.05), and *Cicadoidea* spp. (*X*^2^_3_ = 5.715, *p* > 0.05) (Table 6). 

There were no significant differences in the number of people who have never consumed insects, the people who have consumed insects in their lifetime, and the people who still consume insects (*X*^2^_8_ = 9.041, *p* > 0.05) in KZN. However, there were significant differences in people who have never consumed insects, the people who have consumed insects in their lifetime, and those who still consume insects (*X*^2^_6_ = 13.395, *p* < 0.05) in Limpopo. A greater percentage of respondents reported having consumed at least one insect species in their lifetime in Limpopo (98%) compared to KwaZulu-Natal (64%), with 95% still practicing entomophagy in Limpopo compared to only 28% in KwaZulu-Natal. Thirty-five percent of KZN respondents reported that they used to consume insects (2% who cited that they used to consume insects in the past in Limpopo; the percentage is low because of the high number who currently consume). KZN had a greater number (36%) of respondents who had never consumed insects than in Limpopo (only 3%).

There were significant differences in factors affecting the choice to eat insects in KZN (*X*^2^_15_ = 35.233, *p* < 0.05) and in Limpopo (*X*^2^_19_ = 35.145, *p* < 0.05). In KZN, age (*X*^2^_7_ = 23.764, *p* < 0.05) and educational background (*X*^2^_4_ = 11.208, *p* < 0.05) were the factors that influenced the choice to eat insects, while in Limpopo, employment status (*X*^2^_3_ = 10.913, *p* < 0.05) and gender (*X*^2^_1_ = 3.378, *p* < 0.05) were the factors that influenced the choice to eat insects (Table 7 and Table 8).

### 3.2. Reasons for Consuming Insects, or Not

**Figure 1:**
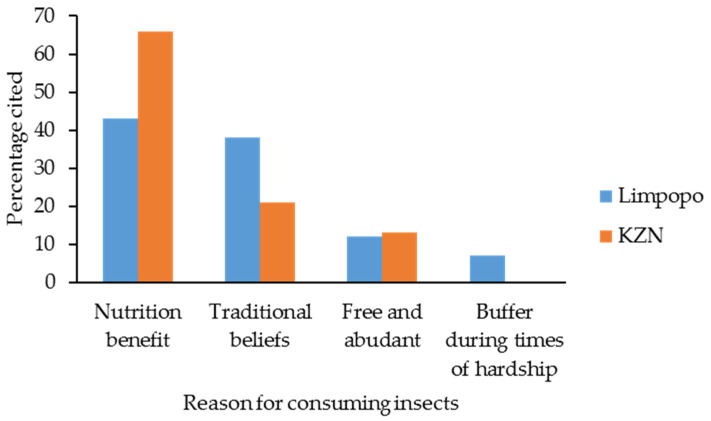
Reasons for consuming insects in Limpopo and KZN provinces of South Africa.

Nutritional benefits of insects and traditional beliefs were the primary reason for practicing entomophagy in KwaZulu-Natal (43% and 38%, respectively) and Limpopo (66% and 21%, respectively) (Figure 1).

**Figure A1:**
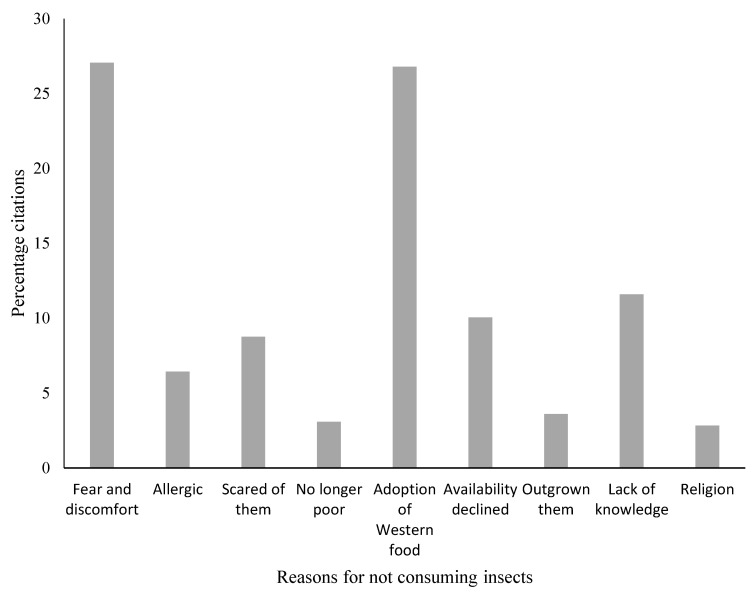
Cited reasons for not consuming insects in KwaZulu-Natal province (*n* = 356).

The most cited reasons (36%) for not consuming insects in KZN were fear and discomfort associated with consuming insects, adoption of western food culture (27%), followed by a lack of knowledge about entomophagy (12%), and a decrease in the availability of edible insects in the wild (10%) (Figure A1). In Limpopo, 3% of the respondents cited religious beliefs as a reason for not consuming insects. In comparison, only 2% cited dislike and discomfort associated with eating insects as a reason for stopping eating insects. Respondents did not include cost as a reason for not consuming insects, because in KZN, insects were collected from the wild, and in Limpopo, insects were also collected from the wild and sold for R20–R30 a cup in town.

There were significant differences in reasons for consuming edible insects among respondents of different genders (*X*^2^_1_ = 6.361, *p* = 0.012) and age group (*X*^2^_7_ = 78.308, *p* = 0.001); however, there were no significant differences in reasons for consuming edible insects among respondents with different educational backgrounds (*X*^2^_4_ = 2.567, *p* = 0.633) and employment status (*X*^2^_4_ = 1.635, *p* = 0.802) in KZN. In Limpopo, there were significant differences in reasons for consuming insects among respondents of different genders (*X*^2^_1_ = 10.215, *p* = 0.001), ages (*X*^2^_1_ = 44.568, *p* = 0.001), and employment status (*X*^2^_3_ = 30.850, *p* = 0.001). However, there were no significant differences in reasons for consuming insects among respondents with different educational backgrounds (*X*^2^_3_ = 4.746, *p* = 0.191).

There were significant differences in reasons for not consuming edible insects among different genders (*X*^2^_1_ = 5.213, *p* = 0.019), ages (*X*^2^_7_ = 15.967, *p* = 0.025), and educational backgrounds (*X*^2^_4_ = 13.525, *p* = 0.009); however, there were no significant differences in reasons for not consuming insects among respondents with different employment status (*X*^2^_4_ = 1.366, *p* = 0.850) in KZN. On the other hand, there were no significant differences in reasons for not consuming edible insects among respondents of different genders (*X*^2^_1_ = 0.078, *p* = 0.780), ages (*X*^2^_7_ = 4.231, *p* = 0.753), educational backgrounds (*X*^2^_3_ = 0.559, *p* > 0.906), and employment status (*X*^2^_3_ = 2.246, *p* > 0.523).

### 3.3. Frequency of Consumption

**Figure A2:**
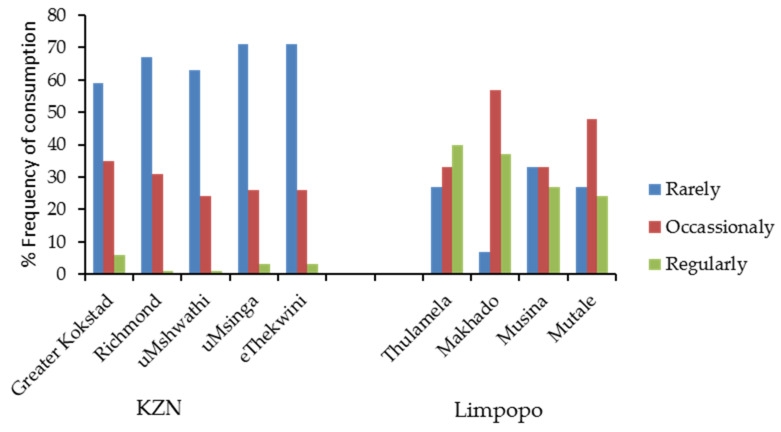
Frequency of insect consumption per week and month across municipalities in KwaZulu-Natal and Vhembe district, Limpopo. Rarely consume insects (at least once a month), occasionally (1–4 times a month), regularly (1–4 times a week).

There were no significant differences in insect consumption frequency across local municipalities in KZN (*X*^2^_4_ = 1.680, *p* = 0. 794). The majority (59–71%) of the respondents rarely consume insects (at least once a month), while fewer respondents (1–6%) consumed insects regularly (1–4 times a month) in KZN (Figure A2). The frequency of insect consumption did not differ significantly (*X*^2^_9_ = 15.317, *p* = 0.083) across local municipalities in Limpopo; 33–57% of the respondents consumed insects occasionally while only 24–40% consumed insects at least once a month (Figure A2).

There were significant differences in frequency of edible insect consumption among different genders (*X*^2^_1_ = 6.980, *p* = 0.008) and ages (*X*^2^_7_ = 70.704, *p* = 0.001); on the other hand, there were no significant differences in frequency of edible insect consumption among respondents with different educational backgrounds (*X*^2^_4_ = 2.970, *p* = 0.563) and employment backgrounds (*X*^2^_4_ = 0.748, *p* = 0.945) in KZN. There were no significant differences in frequency of edible insect consumption among different genders (*X*^2^_1_ = 0.020, *p* = 0.928), ages (*X*^2^_7_ = 0.028, *p* > 0.05), educational backgrounds (*X*^2^_3_ = 0.034, *p* > 0.05), and employment status (*X*^2^_3_ = 0.048, *p* > 0.05) in Limpopo.

### 3.4. Preparation and Collection Methods

Seasonal availability, collection, and preparation methods of edible insects differed from one species to another (Table 4). All respondents collected insects from the wild in KZN, while almost half in Limpopo bought insects from towns (46% from Elim, Sibasa, Louis Trichardt, Makhado-Biaba, and Tshakhuma markets, or Thohoyandou). All the insects consumed are collected from the wild and are not reared for consumption. *Gynanisa* caterpillars and termites were abundant in the rainy season (from October to January), while stink bugs occurred in the dry season from May to August.

Edible insects are mainly collected in the wild mainly by women and they are prepared using different methods (Table 4). They are eaten either fried, boiled, roasted, sun-dried, or as a relish (Table 4). A greater number of respondents preferred sun-dried (62%) edible insects in Limpopo, while fried or roasted edible insects were the most preferred (88%) cooking method in KwaZulu-Natal.

## 4. Discussion

Entomophagy is an important traditional practice in Africa’s different regions [4,46]. The current study reported a greater number of respondents consuming insects in their lifetime in Limpopo (98%) and KwaZulu-Natal (64%). These results are similar to those of Shackleton et al. [32]. They reported that 96.3% of the respondents in Ha-Gondo village, 55 km east of Thohoyandou in Limpopo, used insects as food; by comparison, 67.8% of the respondents in KwaJobe village in northern KwaZulu-Natal used insects as food. These results indicate that entomophagy is a common practice in South Africa. In addition, Egan [49] reported that 90.5% of the respondents consume insects in Blouberg Municipality in Limpopo. According to Teffo et al. [15], the consumption of insects in South Africa is more prevalent in Limpopo, Gauteng, North West, and Mpumalanga provinces. Across African countries, Zimbabwe [4,5,6,7,8,9,10,11,12,13,14,15,16,17,18,19,20,21], Angola [54], Tanzania [36], Nigeria [55], and Botswana [18] have been reported to consume edible insects. However, Ethiopia is one exception, because religious beliefs prohibit the consumption of insects, and only 1% of people interviewed were prepared to accept insects as a human food [56].

However, the consumption of insects may be declining in some areas, as reported in the current study; only 28% still consume insects in KwaZulu-Natal. According to Doberman et al. [57], the consumption of insects seems to be declining because of the spread/adoption of western food choices, and the association of insects with fear and discomfort when it comes to eating them. In addition, the way of living is constantly changing, and is being influenced by modern technology and education, resulting in people neglecting traditional practices that formed part of their lifestyle in the past [50,58]. Documenting indigenous knowledge about edible insects will preserve long-standing traditional knowledge about edible insects that can help influence planning and decision-making on the sustainable use of insects as a nutritious food that can ensure food security for people in developing countries [50,59].

The choice not to eat insects is primarily influenced by preference, availability of insects, and consumer acceptance [60]. Findings from the current study revealed that religion (for example, the “African-initiated church” such as the Zionist churches) and fear associated with consuming insects were the main reasons influencing the decision not to eat insects. According to van Huis [61], the consumption of insects is perceived as unholy, dirty, and unhealthy by some people, particularly in developed countries. For example, Balzan [24] reported that, in Italy, respondents associated insect meals with dirt and food contamination. In addition, Netshifhefhe et al. [31] found that 80.7% of the respondents in Limpopo cited that some religions are against the consumption of insects, especially traditional churches. Culture plays a significant role in determining acceptance and preference of edible insects. For example, *Zonocerous* spp (a grasshopper) is consumed as food in Cameroon, Nigeria, and South Africa, but the same species is considered poisonous elsewhere [62]. In addition, cultural beliefs influence how insects should be prepared before consumption [62]. Religious and gender-based taboos also govern the consumption of insects in some regions. For example, religion and customs prohibit women of the Baganda tribe in Uganda from consuming nsenene (*Ruspolia differens*) [36,62]. There is a need to educate and raise awareness about the consumption of insects through media to promote and encourage people to adopt edible insects as foods, because insects are a sustainable nutritious food which has less impact on the environment [58]. In addition, incorporating insect powder into food products such as bread, biscuits, snack bars, cereal, porridge, and shakes might promote acceptance of insects as food [60].

Sociodemographic factors play a significant role in a person’s choice to consume insects [63]. This study found that gender, age, occupation, and educational background were the main factors influencing the choice to eat insects. These results are similar to Anakware et al. [64], a study which found that gender, age, educational background, and occupation significantly influenced people’s choice to consume insects in Ghana. Youth, educated, urban dwellers, and middle- and upper-class earners are highly influenced by western culture [65]. As a result, they are adopting western diets and ignoring traditional food such as edible insects. There is a notable shift to adopting Western diets, and a decline in consumption of edible insects, particularly by youth and educated people [65]. This might be the main reason why the consumption of insects is largely practiced in rural areas where high levels of unemployment and people with no formal education are found. In addition, people who are unemployed consume edible insects more because they have to do so to meet nutritional requirement needs.

Eight insect species belonging to five insect orders were used as food in Limpopo and KZN. These results are similar to Obopile and Seeletso’s [18] findings, who reported that insects belonging in six insect orders were used as food in Botswana. Contrary to this, Makhado et al. [33] reported that insects belonging in four insect orders were used as food in Greater Giyani Municipality, Limpopo. This suggests that the consumption pattern and preference of insects vary from place to place [66]. According to Raheem et al. [1], insects and preference consumption varies from country to country, and variations can be observed between ethnic groups in different countries. The variation in the number of insects consumed in different countries is attributed to the availability and occurrence of edible insects in the wild [62]. Differences in geographic area and environmental conditions influence the occurrence of different species; for example, mopane worm (*Imbrasia belina*) occurs in mopane woodlands in Southern Africa and is mostly used as food in this region [67].

Mopane worms and termites were the most preferred and consumed insects in Limpopo and KwaZulu-Natal, respectively. Kelemu et al. [27] reported that mopane worms and termites are the most consumed species in Southern Africa. In addition, they are a popular traditional food in many cultures in Southern Africa [31,68]. According to Baiyegunhi et al. [68], mopane worms are occasional delicacies for different cultures in South Africa. Edible insects are valued natural resources that people collect for food and income in rural areas; they are used as a food security safety net in rural areas where poverty and malnutrition are major problems [33,67,68].

This study found that edible insects are consumed because of their nutritional value and they contribute to nutritional diets in rural areas. Similar results were reported by several previous studies [4,15,31]. Netshifhefhe et al. [31] conducted a study looking at the human uses and indigenous knowledge of edible termites in Vhembe district, Limpopo province, South Africa, and found that the majority of the respondents consumed edible termites for their nutrition and to enhance their health. Similarly, Manditsera [4] conducted a study on consumption patterns of edible insects in rural and urban areas in Zimbabwe and found that the primary motives for consuming insects in rural and urban areas were nutrition and taste of edible insects. Food shortage and malnutrition are prevalent challenges experienced in rural communities in Southern Africa [38]. Edible insects play an important role in supplementing diets in poor communities across Africa [46]; in addition, edible insects are used to ease food shortages and provide vulnerable communities with nutritious diets that improve human health and wellbeing [69,70].

Climate change is a global problem that reduces precipitation and increases the extended drought in Southern Africa [71,72]. These changes have resulted in the decline of insect availability in the wild [71,73]. Other factors that might affect the availability of edible insects are different land uses such as clearing of land, development, agriculture, and deforestation [61,70,72]. Ndlovu [72] reported that 40% of the respondents in Zimbabwe cited that the cutting down of mopane trees for fuel use resulted in the decline in mopane worm yields. The decline in the availability of insects affects rural livelihoods and the well-being of the people who depend on insects for food and cash income [61,71]. In addition, this affects the nutrition security of rural populations. Interventions to increase insect yields are required. This can be done by farming and rearing to make insects easily available to people, particularly in vulnerable communities [61,70,71]. In addition, because of their nutritional value, insects can be used as nutritious food alternatives to mainstream animal protein such as pork, chicken, beef, and fish [61,70,71,74].

Insects are mainly harvested in the wild by women. According to Dzerefos et al. [75,76], insects collection and preparation are primarily female-driven tasks, with more than 70% (in the current study) of females involved in insects harvesting in Limpopo province. Insect preparation and processing methods differ from species to species. The important step is removing unpalatable parts and degutting before washing [77,78]. After washing, insects are then boiled or roasted, then sundried to increase shelf life [79,80]. According to Agea et al. [28], sun-drying insects extend the availability of insects and allows traders to have products for a longer period, even when the period of occurrence of insects has passed.

This study reported that edible insects are incorporated in regular diets and they are eaten fried, boiled, roasted, dried, or as a relish. Similar findings were reported by other authors [4,21,31,81]. They found that edible insects are eaten fried, boiled, dried, or as a relish. However, in some parts of Africa, edible insects are smoked or eaten raw without preparation [66,82]. Tradition and culture influence cooking methods of edible insects. People prepare insects based on the knowledge that has been passed down from older generations; for example, in Limpopo province, South Africa, *Encosternum delergorguei* (stink bug) is eaten either fried or raw, while in Zimbabwe, the same species is eaten fried or dried but not raw [15,21]. In addition, some tribes prohibit the consumption of raw edible insects; in Uganda, the Baganda tribe customs and tradition prohibit the consumption of raw *Ruspolia differens* [36]. Cooking methods improve the sensory quality of edible insects through the formation of aromatic compounds [83]. According to Gosh et al. [62], sensory characteristics such as taste, texture, odour, colour, and appearance play an important role in food selection, acceptability, and preference. Some cooking methods reduce foodborne and degradative enzymes, which increase the shelf life of edible insects [83].

This study’s limitations were that local communities refer to several species from one insect order/genus using the same common vernacular names. Therefore, there might be an underrepresentation of the total number of species consumed in the two provinces. Respondents relied on the pictures from the insect guidebook to identify the correct species they consume; species that were not represented in the book might have been left out of the survey.

## 5. Conclusions

Edible insects play a crucial role in food and nutrition security in rural communities of Limpopo and KwaZulu-Natal provinces, South Africa. This study recorded eight species belonging to five insect orders that are used as food in these provinces. Indigenous knowledge about the collection and preparation of edible insects needs to be preserved, because it can play an important role in the promotion of edible insects as food. Entomophagy is still practiced in Limpopo and KwaZulu-Natal. However, there is a notable decline in the availability of edible insects and the consumption of insects in KwaZulu-Natal. This is thought to be a result of the adoption of western food culture, religion, fear/discomfort associated with eating insects and the decline in the availability of insects in the wild.

The latter is concerning because people are losing critical traditional practices that play a major role in rural nutrition and livelihoods. Edible insects are highly nutritious and have a lower environmental impact than livestock production [5]. To promote entomophagy, there is an urgent need for education and awareness about edible insects and their benefits to help reduce the stigma, fear, and discomfort associated with eating insects. In addition, documenting indigenous knowledge about the consumption of edible insects in media and literature will help promote edible insects, ensure that indigenous knowledge about edible insects is preserved, and change peoples’ perceptions about edible insects. In addition, indigenous knowledge about entomophagy will contribute to local, national, and global knowledge about edible insects, which might help guide the inclusion of edible insects in food policy, enabling the adoption of insects as food that will be included in daily diets and to better understand edible insects as a potential solution to food security problems, particularly in developing countries [49,50]. Future research should focus on the nutritional content of edible insects, and the potential of farming and rearing of edible insects in South Africa to increase the availability of edible insects, and make them easily accessible to people. Government officials should encourage people, especially from vulnerable groups, to include edible insects in their daily diets. To promote acceptability, edible insects could be incorporated into food products. In addition, more research should focus on the acceptability of food products fortified with edible insects.

## Figures and Tables

**Table 1:** Population size of local municipalities surveyed in KwaZulu-Natal (KZN) and Limpopo provinces.

Municipalities	Population Size
Limpopo	
Makhado	416,728
Thulamela	497,237
Mutale	91,870
Musina	32,009
KZN	
uMshwathi	10,374
Richmond	65,793
uMsinga	160,000
Greater Kokstad	51,561
eThekwini	3,702,231

adapted from [51,52].

**Table 2:** Demographic information of the respondents in five local municipalities, KwaZulu-Natal (*n* = 500), South Africa.

Demographics	KZN Overall (%)	Greater Kokstad	Richmond	UMshwathi	uMsinga	eThekwini
**Age category (years)**						
Under 18	8	9	8	9	9	6
18–24 years	18	13	9	24	23	23
25–34	15	19	22	10	13	11
35–44	10.6	14	9	7	11	12
45–54	11.2	10	10	12	11	13
55–64	15.8	15	23	15	15	11
65–74	11.8	12	13	13	8	13
75 years and above	9	8	6	10	10	11
Sex						
Male	40	41	40	42	40	37
Female	60	59	60	58	60	63
**Level of education**						
No formal education	23	20	27	14	26	30
Primary education	37	39	44	34	33	34
Secondary education	32	26	26	44	32	33
Tertiary education	8	15	3	8	9	3
**Occupation**						
Unemployed	32	30	35	22	28	46
Self employed	11	9	15	12	13	6
Pensioner	32	34	37	34	25	29
Employed	15	18	8	19	21	10
Student	10	9	5	13	13	9

**Table 3:** Demographic information of the respondents in four local municipalities in the Vhembe district, Limpopo province (*n* = 122).

Demographics	Limpopo Overall (%)	Thulamela	Makhado	Musina	Mutale
**Age category (years)**					
Under 18	9	0	9	1	1
18–24 years	16	7	6	4	2
25–34	23	16	7	4	2
35–44	12	5	6		4
45–54	13	11	4		1
55–64	13	8	4		2
65–74	11	9	4		2
75 years and above	4	3			2
**Sex**					
Male	48	28	18	5	7
Female	52	32	19	4	9
**Level of education**					
No formal education	7	7	1	1	
Primary education	14	11	2		4
Secondary education	57	24	32	6	8
Tertiary education	22	6	16	1	3
**Occupation**					
Unemployed	60	30	25	8	10
Self employed	18	13	6		3
Pensioner	17	11	7		3
Employed	5	3	2	1	

**Table 4:** Traditional collection and preparation methods of the commonly consumed insects in KZN and Limpopo provinces of South Africa.

Insects Group	Insect Order	Consumption Stage	Seasonality	Method of Collection	Collectors	Processing for Preservation Method	Cooking Method
*Gynanisa* caterpillar	Lepidoptera	Larvae	November–January	Collected from the host plant	Women	Degutted, washed, boiled in salt water, and sun-dried	Fried, roasted, or as a relish
Mopane worms (*Imbrasia belina*)	Lepidoptera	Larvae	Nov–January; April–May	Collected from *Colophospermum mopane*	Women	Degutted, washed, boiled in salt water, and sun-dried	Fried, boiled without salt, boiled with salt, roasted, or as a relish
Termites (*Macrotermes* species)	Blattodea	Winged adult	September–January	Trapped in a large bowl of water near the light source	Women	Killed with boiling water, boiled, and sun-dried	Fried and roasted
Stinkbug (*Encosternum delegorguei*)	Hemiptera	Adult	May–August	Picked from woodlands	Women	Killed with warm water, cooked, and dried	Fried, roasted
*Cirina forda*	Lepidoptera	Larvae	Nov–Feb	Picked from the host tree	Women	Degutted, washed, boiled in salt water, and sun-dried	Fried, boiled, boiled with salt, roasted, or as a relish
Edible grasshopper/locust (*Locustana and Zonocerous* species)	Orthoptera	Adult		Picked from grassland	Women and children	Dewinged, degutted, killed in hot water and roasted	Fried, roasted
*Carebara vidua*	Hymenoptera	Adult	All year round	Picked from grassland	Women and children	Eaten raw	Eaten raw
*Cicadoidea* spp.	Hemiptera	Adult		Picked from grasslands		Killed with warm water, cooked, and dried	Fried, roasted

**Table 5:** Percentage of respondents that consumed insects across five municipalities in KZN, South Africa.

Insects	Greater Kokstad	Richmond	uMshwathi	uMsinga	eThekwini	Average
Termites	74	72	65	67	70	70
Edible locust	6	6	34	40	19	22
*Cirina forda*	0	1	13	19	2	8

**Table 6:** Percentage of respondents that consumed insects across four municipalities in Vhembe district in Limpopo, South Africa.

Insects	Thulamela	Makhado	Musina	Mutale	Average
Mopane worm	100	97	90	97	96
Termites	97	97	83	70	86
*Gynanisa* caterpillar	16	13	20	10	14
Stink bug	16.6	16.6	30	13	19
Edible grasshopper	97	97	76	73	85
*Carebara* spp.	16.6	13.3	20	13.3	15
*Cicadoidea* spp.	0	0	6.6	0	2

**Table 7:** Factors affecting choice to consume insects in KZN, South Africa.

Factors	Chi-Square	df	*p* Value
Gender	1.080	1	0.299
Age	23.765	7	0.001
Educational background	11.208	3	0.011
Occupation	4.662	4	0.324

**Table 8:** Factors affecting choice to consume insects in Limpopo, South Africa.

Factors	Chi-Square	df	*p* Value
Gender	3.738	1	0.053
Age	6.748	7	0.456
Educationalbackground	2.014	3	0.570
Occupation	10.913	4	0.002

## Data Availability

The data presented in this study are available in the tables, figures as well as appendices of the current manuscript.

## Appendix A

**Table A1:** Number of villages sampled in different local municipalities in Limpopo and KwaZulu-Natal.

Village	Local Municipality	GPS Coordinates
	**Limpopo province**	
Muwaweni	Makhado	23.3283° S, 30.1113° E
Elim	Makhado	23.1561° S, 30.0554° E
Biaba	Makhado	22.5344° S, 30.1126° E
Tshikuwi	Makhado	22.9013° S, 29.9480° E
Tshiozwi	Makhado	23.0855° S, 29.7860° E
Ha Mashau Doli	Makhado	23.1583° S, 30.1897° E
Ha mashau Mathothe	Makhado	23.1462° S, 30.1979° E
Masia	Makhado	23.2170° S, 30.3299° E
Tshikota	Makhado	23.0492° S, 29.8772° E
Mashamba	Makhado	23.0538° S, 30.3527° E
Mulima-Lambani	Makhado	22.430° S, 30.500° E
Nancefield	Musina	22.3813° S, 30.0319° E
Mbodi tshafhasi	Mutale	22.5433° S, 30.7451° E
Shakadza	Mutale	22.6160° S, 30.5711° E
Tshipise	Mutale	22.5349° S, 30.6703° E
Mukondeni	Makhado	23.2559° S, 30.1041° E
Tshivhulani	Thulamela	23.1316° S, 30.4386° E
Vhurivhuri	Thulamela	22.7008° S, 30.8056° E
Halambani	Thulamela	22.7108° S, 30.8442° E
Ha-Lambani	Thulamela	22.7108° S 30.8442° E
Mushiru	Thulamela	22.7662° S, 30.8431° E
MukulaVyeboom	ThulamelaMakhado	22.8628° S, 30.5754° E 23.1488° S, 30.3861° E
	**KwaZulu-Natal province**	
Umbumbulu	eThekwini	29.5902° S, 30.4207° E
Nhlazuka	Richmond	29.3759° S, 30.100° E
Swayimane	uMshwathi	29.4878° S, 30.6603° E
Tugela Ferry	Umsinga	28.7416° S, 30.4617° E
Kokstad	Greater Kokstad	30.5096° S, 29.4063° E

## Appendix B. Questionnaire Used to Source Information in Limpopo and KwaZulu-Natal

Project Tittle: Diversity of Edible Insects and Their Related Indigenous Knowledge by Human in Limpopo and KwaZulu-Natal Province, South Africa

You are being invited to consider participating in a study that involves research on the consumption of edible insects using indigenous knowledge in South Africa, we would like to get insight on the socio-economic benefits, drivers of consuming edible insects and the attitude people have towards edible insects. The aim of this research is to determine diversity and distribution of edible insects using indigenous knowledge in South Africa. Please note that your participation in this research is voluntary and you may choose not to participate or you may discontinue your participation at any time without penalty or disadvantage to yourself.

In case there are questions considering the survey, please contact Zabentungwa Hlongwane (0737076460 or email nolwazihlongwane20@gmail.com)

Awareness and perception

Date: ____________________________________

Questionnaire number: ____________

Location:__________________________________

Has the informed consent being signed by the participant Yes [ ]/No [ ]

**Questionnaires**

1. Gender Male [ ] Female [ ]
2. Age category
  ▪ Under 18 years [ ]
  ▪ 18–24 years [ ]
  ▪ 25–34 years [ ]
  ▪ 35–44 years [ ]
  ▪ 45–54 years [ ]
  ▪ 55–64 years [ ]
  ▪ 65–74 years [ ]
  ▪ 75 or older [ ]
3. Level of education
  ▪ Level 0 [ ]
  ▪ Primary school [ ]
  ▪ High school [ ]
  ▪ Undergraduate [ ]
  ▪ Post graduate [ ]
4. Employment status: unemployed [ ] self-employed [ ] pensioner [ ] employed [ ]
5. Have you ever eaten insects? Yes [ ] or No [ ] if No go to question number 15
6. If yes how often do you consume insects
  ▪ Rarely [ ]
  ▪ Occasionally [ ]
  ▪ Regularly [ ]
7. How do you prepare and eat edible insects
  ▪ Dried [ ]
  ▪ Fried [ ]
  ▪ Stew (socked in clean water for 30 min, drain water, fried with onions, tomatoes, chili, curry powder and salt) [ ]
  ▪ Please share your recipe of how you cook edible insects for consumption
    ___________________________________________________________________________
    _______________________________________________________________________________________________________________________________________________________________________________________________________________________________________________________________________________________________________________________________________________________________________________________
  ▪ How did your mother cook edible insects when you were growing up
    _____________________________________________________________________________________________________________________________________________________________________________________________________________________________________________________________________________________________________________________________________________________________________________________________________________________________________________________________________________________________________________________________________________
8. Where do you buy or harvest edible insect
  ▪ Local markets [ ]
  ▪ City markets (Thohoyandou, Sibasa, Elim, Makhado or _________________________
  ▪ Harvest from the wild [ ]
9. Why do you consume insects
  ▪ Nutritious benefits [ ]
  ▪ Cultural beliefs [ ]
  ▪ Cheap and abundantly available [ ]
  ▪ Buffer during times of food shortage [ ]
  ▪ All of the above [ ]
10. How long have you been consuming insects ___________________________
11. How many orders of insects are edible in your area ________________________
12. What season are edible insects mostly available
  ▪ Spring [ ]
  ▪ Summer [ ]
  ▪ Autumn [ ]
  ▪ Winter [ ]
  ▪ Available all year [ ]
13. There are many benefits of consuming insects, which of the following interest you the most?
  ▪ Environmental friendly [ ]
  ▪ Nutritional value [ ]
  ▪ Cheaper [ ]
  ▪ Taste [ ]
  ▪ None of the above [ ]
14. Would you ever consider eating insects? Yes [ ] or No [ ]
15. Do you know anything about edible insects. Yes [ ] or No [ ]
16. What can be done to promote eating of insects.
  ▪ Research [ ]
  ▪ Public awareness [ ]
  ▪ Sell them in shops [ ]
  ▪ Newspapers [ ]
  ▪ Radio and television [ ]
  ▪ Education and awareness [ ]
17. Are you aware of the nutritional benefits of eating insects yes [ ] or no [ ]
18. Would you be more comfortable eating insects if there were hidden in food? (covered in chocolate, cookies) yes [ ] or no [ ]
19. Which insect do you consume or would you consider eating
  ▪ Mopane worms [ ]
  ▪ Crickets [ ]
  ▪ Termites [ ]
  ▪ Locusts [ ]
  ▪ Grasshoppers [ ]
  ▪ Ants [ ]
  ▪ Beetles [ ]
  ▪ Mealworms [ ]
  ▪ Stink bug
20. Why did you stop eating insects?
  ____________________________________________________________________________________________________________________________________________________________
  ______________________________________________________________________________
21. Is there anything you would like to tell us regarding the eating of insects by human?
  ________________________________________________________________________________________________________________________________________________________________________________________________________________________________________________________________________________________________________________________________________________________________________________________________________________________________________________________________________________________________________________________________________________________________________________________________________________________________________________
